# Advancing Dementia Care: AI Simulations in Managing Everyday Challenges

**DOI:** 10.1002/alz.093188

**Published:** 2025-01-09

**Authors:** Fengpei Yuan, Bryce Bible, Xiaopeng Zhao

**Affiliations:** ^1^ University of Tennessee, Knoxville, TN USA

## Abstract

**Background:**

The prevalence of Alzheimer’s disease and related dementias (ADRD) presents a growing public health challenge. This study introduces an innovative approach to dementia care through the development of AI agents that simulate the interactions between people living with dementia (PLWD) and their caregivers during activities of daily living (ADLs).

**Method:**

The study employs ChatGPT (GPT4) large language models to create AI agents representing both PLWD and interventionists. These agents are designed to mimic real‐world verbal and nonverbal interactions in a simulated environment. A key component of the research is the development of a reinforcement learning (RL) framework. This framework is instrumental in formulating optimal assistive strategies that are personalized for PLWD. The performance of the RL‐guided interventionists is compared with that of naive interventionists across various scenarios. The focus is on tasks such as grocery shopping, highlighting the potential challenges and assistance required in such daily activities.

**Result:**

The AI agents successfully replicated the nuanced dynamics of dementia care. The RL framework demonstrated its potential in optimizing personalized care strategies, highlighting the significant role of AI in dementia management. Comparisons revealed that RL‐guided interventionists outperformed their naive counterparts, indicating the effectiveness of AI in enhancing caregiving approaches. The study also emphasizes the need for future research to include more complex scenarios and integrate real‐world data to further enhance the simulation’s accuracy and relevance.

**Conclusion:**

This research represents a groundbreaking step in applying AI to dementia care. It showcases the capability of AI agents in understanding and replicating complex human interactions within the context of caregiving. The study opens new pathways for research in AI’s role in healthcare, especially in addressing the challenges of eldercare and dementia management. It suggests a future where AI can not only assist in understanding and managing dementia but also support caregivers, thereby improving the quality of life for both PLWD and those who care for them. The integration of professional expertise and real‐world data in future studies could further refine these AI‐based care strategies, making them more effective and personalized.

